# Employment of Nitric Acid and Opium for the Treatment of Autumnal Forms of Diarrhœa and Dysentery

**Published:** 1862-04

**Authors:** Patrick J. Hynes

**Affiliations:** Nottingham


### EMPLOYMENT OF NITRIC ACID AND OPIUM

### FOR THE TREATMENT OF THE AUTUMNAL FORMS OF DIARRHŒA AND DYSENTERY.

By PATRICK J. HYNES, M. D., Nottingham.

The periodical recurrence of these well-known visitants is so certain as to become almost "as familiar as household words;" in fact, to such an extent is this the case, that medical men in extensive practice, enjoying, as it were, an interval of comparative repose during the summer months, from the fatigue consequent upon their attention to the host of diseases of the pulmonary organs inevitable in the cold and dampness of our climate, buckle on their armor to combat, each in his own peculiar fashion, with an enemy which is, if we may judge from the present rate of mortality in the metropolis, (*vide* the Registrar General's "Return" of the 17th inst.,) of an equally formidable character.

I am not going to write an elaborte article upon the above diseases, since the numerous treatises that abound describe the various stages and symptoms of these complaints; but my object is to bring *more prominently* forward a remedy, which, although it is by no means a new one, still I think is not generally employed; and I am bold enough to hope that its more extensive use in the hands of the medical gentlemen of the metropolis would have the effect of mitigating much of the present rate of mortality. About ten year since diarrhœa and dysentery prevailed rather extensively in this locality, and emboldened by the successful treatment of these diseases from the remedy I was in the habit of employing, I ventured to bring it, through the medium of the public journals, into notice, and since that time I have been repeatedly assured by many gentlemen who adopted it that my opinion of its efficacy has been by no means exaggerated. I believe I am indebted to the works of Dr. Abercrombie, "On Diseases of the abdominal Viscera," for the formula I am in the habit of employing; but as I write from memory, and have not an opportunity of confirming my opinion on this point by a direct reference to that excellent work, I do not wish to deprive any other author of whatever merit may be found to attach to its introduction as a remedy for those diseases.

I may observe that during the last four or five weeks diarrhœa has been very prevalent in this locality, and I do not overstate when I assert that I have used it in more than a hundred cases already, and always with almost instantaneous benefit. The formula that I generally employ is as follows: Compound infusion of gentian, eight ounces; tincture of opium, a drachm to a drachm and a half; nitric acid, twenty minims; one ounce to be taken after every liquid stool or painful alvine evacuation. A mustard plaster applied to the epigastrium, and drinking sparingly of ice-cold mint-tea, relieve the sickness and thirst that frequently accompany the severer form of these diseases. I have found the remedy equally beneficial in some rather intractable form of dysentery. I would strongly urge upon gentlemen, who meet with dysenteric cases, to give it a trial, as I feel satisfied they will find it a very powerful curative agent in their hands.

In an old number of the *Medical and Physical Journal*, from which I several years since made an extract, I find the following from a Mr. Hope, of Chatam, He says:

"The first occasion of its use was remarkable. A young man of sobriety and temperance, had suffered long with dysentery, and had been attended by a friend of mine for some time, who recommended those remedies that high authority and experience pointed out for relief; but finding no advantage, he sent for me, expecting more might be done. Nothing, however, recommended was successful, and, as I could but go over the same ground, no prospect of relief appeared; indeed his death was daily expected. At this time a woman who lived with him was attacked with dysentery, with extreme thirst. An acid occurred to me; but, fearing that it might produce unpleasant effects, opium was added:—Nitric acid, two drachms; opium, two grains; water, two ounces; a spoonful to be taken in any vehicle three or four times daily. The effect produced was so great that my dying patient, unknown to me, begged to partake with her, and when I saw him next morning, which to my great surprise was with a cheerful countenance, he told me if ever I had a patient ill with his complaint, I should never fail to send the drops I had sent for the woman, for they had relieved his complaint at the first dose, and he was sure he should mend now, for they had saved his life this time.

This was the only medicine he took, and in a few days he was able to walk about his room. In a third case I tried the acid without the opium, but it did not succeed. I then united them and it effected immediate relief. I was still unwilling to persuade myself into a belief of its being a specific remedy, until a case of so extraordinary a nature occurred as compelled me to decide unequivocally in its favor. A young lad, sixteen years of age, fell over a dredging-boat into the water, in the month of July last. Indisposition succeeded, but to what degree I could not determine, as another practitioner was at first called in to his assistance. This was near a month after the accident. The remedies I applied failed in their efficacy. His friends, dispairing of relief, requested me not to trouble myself to attend him any more (they lived six miles from me) saying he must be left to his fate, being assured a day or two more would finish the scene. At this time I recommended the anodyne, and with great difficulty it was that they could be persuaded to give it a trial. Twenty-four hours had not elapsed before he began to find relief, so that in four hours he let off the remedy. The disease returned; reapplication of the drops again removed it, and in a very short space of time, without any other medicine intervening, he became as healthy as he ever had been. The manifest advantage of the medicine was recognized by the parents; nor did they spare unjust reflections that I did not employ it before."

I am unwilling to theorize upon the *methodus medendi* of what I believe to be the principal agent in the above formula; yet I cannot help thinking that the nitric acid possesses some disinfecting agency, no less than an astringent efficacy, over autumnal diseases. "The fumes of nitric acid are believed to be efficacious," says the late Dr. Montgomery, "in destroying the effluvia of typhus and other febrile diseases." Diluted with water so as to form an assidulous drink. Dr. Duncan of Edinburgh used to employ it in the low fevers that prevailed in the suburbs of that town. But independent of its chemical action over animal effluvia, it appears to me to act as a direct astringent in all the diseases of the mucuous membrane. Thus in purulent opthalmia, what remedy is so efficacious as nitrate of silver in solution? In fact, in all mucuous discharges it is almost the sole remedy upon which the surgeon depends; and of course the nitric acid is the chief agent in this valuable therapeutic. I am in the habit of employing a formula of a nearly similar kind as a tropical application, but with double the amount of acid, in cynanche and in diptheria; and I can speak very strongly upon its beneficial effects. In brokendown constitutions impaired by mercury, by syphilis or other irregularities, the above remedy will be found frequently valuable; and given in combination with taraxacum, it will prove very serviceable in sluggish conditions of the liver. I am in the habit of employing it with very considerable advantage in the diarrhœa of infants, and, combined with the muriated tincture of iron, in tabes mesenterica. I may further state that I have given the other acids, singly and in combination, the full benefit of a fair trial in all the above forms of diseases; and, without any prepossession or prejudice, my experience enables me to give a decided preference to the claims of the nitric acid in combination with opium, as a very superior therapeutic remedy. I have not had an opportunity of making trial of it in the the very severe forms of epidemic cholera, although I have had ample opportunities of treating that disease. I have, however, many years ago, tried extensively, *inter alia*, the plan of treatment recommended by Dr. Toulmin, of Brighton, thus anticipating his views (see the *Lancet* for Nov., p. 301), which certainly look well in print, particularly as they are eloquently, indeed elegantly, introduced; but when weighed in the balances they will be found wanting.

In conclusion I may observe, that the large mortality which these diseases bring in their train calls for all the curative agency the profession can muster to encounter their fatality. I am vain enough to indulge the hope that more extensive employment of my suggestions may not be altogether useless; and, in the unassuming language of the Roman writer shall say—

> " Si quid novisti rectius istus
> Candidus imperti, si non his uteria mecum."

—*London Lancet.*

